# mSphere of Influence: Forgotten Questions

**DOI:** 10.1128/mSphere.00520-21

**Published:** 2021-06-23

**Authors:** Laura-Isobel McCall

**Affiliations:** a Department of Chemistry and Biochemistry, University of Oklahoma, Norman, Oklahoma, USA; b Department of Microbiology and Plant Biology, University of Oklahoma, Norman, Oklahoma, USA; c Laboratories of Molecular Anthropology and Microbiome Research, University of Oklahoma, Norman, Oklahoma, USA

**Keywords:** chemical cartography, disease tolerance, mSphere of Influence, metabolomics, patient needs, tropism

## Abstract

Laura-Isobel McCall studies the relationship between location and disease pathogenesis, with a focus on infectious diseases and neglected diseases of poverty. In this mSphere of Influence article, she reflects on how three papers, “Opposing effects of fasting metabolism on tissue tolerance in bacterial and viral inflammation” (A. Wang, S. C. Huen, H. H. Luan, S. Yu, et al., Cell 166:1512−1525.e12, 2016, https://doi.org/10.1016/j.cell.2016.07.026), “Three-dimensional microbiome and metabolome cartography of a diseased human lung” (N. Garg, M. Wang, E. Hyde, R. R. da Silva, et al., Cell Host Microbe 22:705−716.e4, 2017, https://doi.org/10.1016/j.chom.2017.10.001), and “‘It’s like a phantom disease’: patient perspectives on access to treatment for Chagas disease in the United States” (C. J. Forsyth, S. Hernandez, C. A. Flores, M. F. Roman, et al., Am J Trop Med Hyg 98:735−741, 2018, https://doi.org/10.4269/ajtmh.17-0691), shaped her spatial approach to infectious disease pathogenesis and helped her broaden her perspective from a pathogen-centric focus to a holistic view that include diseases tolerance mechanisms and barriers to health care access.

## COMMENTARY

We always tend to think that scientists ask “How?” or “Why?”. Sometimes “Who?” and “When?” (“Who is susceptible to an infection?” “When did this pathogen emerge?,” in a microbiology context). Often, the forgotten question is “Where?”. Where is disease happening on a global scale? Where is it happening within a body? In which organs and organ subregions? Location is at the center of infectious disease pathogenesis, from understanding which tissues are colonized by a pathogen (pathogen tropism) to understanding the location of disease symptoms (disease tropism). Being able to answer these “where” questions is fundamental to understand why some infections are more lethal than others, why some people fail to recover from infections, and how we can develop new treatments for infectious diseases.

Tropism (“Where?”) has been the driving question behind my entire research career. Initially, I focused only on the pathogen, investigating the virulence factors that distinguish dermotropic from viscerotropic *Leishmania* parasites and how nutrient availability for the parasite might shape Trypanosoma cruzi tissue distribution (e.g., reference 1). Building on these results, I discovered that treatment with the amino acid derivative carnitine improves acute-stage survival of T. cruzi*-*infected mice by selectively restoring cardiovascular metabolism, in the absence of effects on pathogen load (2). This observation led me to the disease tolerance literature. Disease tolerance encompasses the mechanisms that lead to improved disease outcomes, without reducing pathogen load. Oftentimes, disease tolerance mechanisms may reduce infection-associated or immune response-mediated tissue damage. Disease tolerance is understudied compared to mechanisms associated with pathogen killing (resistance).

While reading about these topics, I encountered the first paper that I wish to highlight, by Ruslan Medzhitov’s group (3). In this paper, Wang et al. demonstrate how modulation of glucose metabolism can switch the outcome of an infection. In the case of Listeria monocytogenes infection, glucose supplementation reduced survival, with only minor increases in L. monocytogenes levels in the spleen, and no impact on liver *Listeria* levels. In contrast, glucose supplementation increased survival during influenza virus infection, while inhibition of glycolysis had antisurvival effects, in the absence of changes in viral load or immune cell infiltration. The lack of effects of these metabolic modulators on pathogen load indicate tolerance-modulating mechanisms. Furthermore, these contrasting findings are an important demonstration that disease tolerance mechanisms are context specific, tied to the nature of the initial pathogenic insult and the corresponding necessary response mechanisms. Last, the mechanism of action involved effects on the brain rather than the lung, indicating the importance of a spatial perspective and of looking beyond standard sites of tissue damage in disease tolerance studies.

A second major influence for me was the development by Pieter Dorrestein’s laboratory of new methods to quantify and visualize the spatial distribution of small molecules (metabolites). One particular study by Garg et al., which inspired me when I first heard its results from P. Dorrestein at a local seminar, mapped the distribution of small molecules and bacteria in the explanted lung from a cystic fibrosis patient (4). This method enabled the study of spatial patterns of Pseudomonas quorum sensing and rhamnolipid metabolites in the lung and of the differential lung distribution of inhaled versus intravenous drugs. Combining drug mapping versus bacterial mapping demonstrated anticorrelation of the antibiotic meropenem and *Achromobacter*. I immediately saw the potential of this method to understand pathogen tropism and antiparasitic treatment failure. Implementation of this mapping approach to my interests in parasitic disease tropism directly led to my findings on metabolites correlated and anticorrelated with T. cruzi distribution in the heart (5) and on the localized nature of metabolic perturbation in Chagas disease (2, 6).

A third major influence was work by S. K. Meymandi and the Center of Excellence for Chagas disease on Chagas disease patient management and on patient perspectives (7). A significant proportion of my postdoctoral research efforts were focused on high-throughput screening to identify new bioactive compounds against parasitic agents, and my research group continues to investigate how our spatial maps of infectious diseases can help guide the development of new interventions. However, this paper by Forsyth et al. highlights how access to treatment and its costs remain a major barrier for patients. It reminds us all that the identification of new treatment regimens is only the first step and that any such research must consider the needs of the patient population and a path to the clinic.

Garg et al.’s paper (4) inspired me to learn metabolomics techniques and to use them to understand the spatial aspects of host-parasite-microbiome chemical interactions. Wang et al.’s paper (3) inspired me to look beyond the pathogen, to disease tolerance mechanisms, and to consider ways in which metabolism might contribute to disease tolerance in a spatially resolved fashion. Last, Forsyth et al.’s paper (7) inspires me to use this knowledge to find new ways to address neglected diseases that are responsive to patient needs, so that my research can have a public health impact. The forgotten questions are not just “Where is the pathogen?,” “Where are symptoms developing?,” but also “Where are the patients?” and “What do they need?”.
